# Circadian Responses to Light-Flash Exposure: Conceptualization and New Data Guiding Future Directions

**DOI:** 10.3389/fneur.2021.627550

**Published:** 2021-02-11

**Authors:** Kwoon Y. Wong, Fabian-Xosé Fernandez

**Affiliations:** ^1^Department of Molecular, Cellular, & Developmental Biology, University of Michigan, Ann Arbor, MI, United States; ^2^Department of Ophthalmology & Visual Sciences, University of Michigan, Ann Arbor, MI, United States; ^3^Department of Psychology, BIO5 Research Institute, University of Arizona, Tucson, AZ, United States; ^4^Department of Neurology, McKnight Brain Research Institute, University of Arizona, Tucson, AZ, United States

**Keywords:** light, circadian, rhythms, photostimulation, flash, retina, photoreceptors, ipRGC

## Abstract

A growing number of studies document circadian phase-shifting after exposure to millisecond light flashes. When strung together by intervening periods of darkness, these stimuli evoke pacemaker responses rivaling or outmatching those created by steady luminance, suggesting that the circadian system's relationship to light can be contextualized outside the principle of simple dose-dependence. In the current review, we present a brief chronology of this work. We then develop a conceptual model around it that attempts to relate the circadian effects of flashes to a natural integrative process the pacemaker uses to intermittently sample the photic information available at dawn and dusk. Presumably, these snapshots are employed as building blocks in the construction of a coherent representation of twilight the pacemaker consults to orient the next day's physiology (in that way, flash-resetting of pacemaker rhythms might be less an example of a circadian visual illusion and more an example of the kinds of gestalt inferences that the image-forming system routinely makes when identifying objects within the visual field; i.e., closure). We conclude our review with a discussion on the role of cones in the pacemaker's twilight predictions, providing new electrophysiological data suggesting that classical photoreceptors—but not melanopsin—are necessary for millisecond, intermediate-intensity flash responses in ipRGCs (intrinsically photosensitive retinal ganglion cells). Future investigations are necessary to confirm this “Cone Sentinel Model” of circadian flash-integration and twilight-prediction, and to further define the contribution of cones vs. rods in transducing pacemaker flash signals.

## Introduction

The retina integrates light signals detected across a tripartite network of photoreceptors to convey time-of-day information related to the Earth's rotation and solar cycle directly to the brain's circadian pacemaker, the suprachiasmatic nucleus (SCN) (1–5). Grounded within the crossroads of this light-detection system is a subset of intrinsically photosensitive retinal ganglion cells (ipRGCs) containing the vitamin A-based photopigment, melanopsin (6–14). ipRGCs are recurrently configured in the eye. By virtue of melanopsin expression, these cells are themselves photo-excitable and can operate as independent relays to the SCN, but—nevertheless—also receive synaptic connections from rods and cones (15–24), and participate in a centrifugal feedback pathway involving several types of retinal amacrine interneurons (25, 26). As an emergent unit, the whole of the retinal circuitry that comprises the (non-image forming) input to the SCN is extremely flexible in its reading of ambient light across various intensities, spectra, and patterns of contrast. While it is appreciated that all photoreceptor classes make contributions to irradiance detection and are activated across an overlapping range of wavelengths (27–31), there is particular specialization with regards to each's role in signaling contact with steady (i.e., non-flickering) vs. intermittent light.

For example, ipRGCs provide a sustained signal to the SCN throughout the duration of exposure to a discrete light stimulus lasting up to several hours (32–38); cones, on the other hand, amplify signaling only at the beginning of the exposure owing to their transient response within the first <1 s of light-onset (39–44). This functional dissociation is evident in electrophysiological retinal recordings of ipRGCs (8, 17, 19), single-unit recordings of SCN neurons (33, 41), as well as behavioral comparisons between rodents with selective loss of cones vs. wildtype animals. In the latter case, cone-deficient mice exhibit full-magnitude phase shifts to 15-min but not 1-min light administration (480 nm) (40). Conversely, cone-activating light fails to phase-shift the rodent pacemaker as a *continuous* 15-min stimulus but does so when presented *intermittently* along 15 separated 1-min steps over an hour (42). These aggregated data suggest a wider truth about the circadian pacemaker's timekeeping estimates. They are based on two superimposed changes in incident light: (1) the slow intensity variation of sunlight that marks the day's movement through the morning and afternoon and that which separates the day from the night (~10–12 h; weighted toward melanopsin function); and (2) the higher-frequency changes in irradiance and spectrum that punctuate twilight interludes at dawn/dusk (~30–60 min; weighted toward cone function).

The pacemaker's phase responses to the same light stimulus (e.g., a 15-min pulse) change systematically across the subjective night along a sigmoidal-like wave (45). In the vast majority of species that have been surveyed, light administration in the first half of the night will produce phase delays in behavioral-physiological rhythms commensurate with the difference in timing between the photic stimulation and the timing of dusk in the solar cycle or lights-out within an indoor light-dark schedule [e.g., in humans, lab rodents and *Drosophila*, introduction of the light stimulus 2 h after subjective sunset will delay rhythms by up to 2 h; (46–48)]. The reciprocal is observed in the second half of the night, where light administration will advance rhythms in proportion to how much earlier the light was seen with respect to expected sunrise [e.g., stimulation 2 h before sunrise or lights-on will fast-forward the onset of diurnal physiology and behavior by up to 2 h; (46–48)]. When describing the circadian pacemaker's *phase response curve* (PRC) to light, many commentators note the technical shape of the PRC in passing or the relationship it might bear to a biological phenomenon of interest. Few point out the bigger picture: the circadian PRC to light is arguably the most demonstrable example of the brain's prediction coding.

Predictive processing is a mature field of inquiry in psychology and cognitive neuroscience (49–52), where diverse methodologies have established the brain as a prospection device that interprets sensory information with the express purpose of generating expectations—and thereby obtaining a level of preparedness—for the immediately relevant future (49–61). Early studies of prediction coding or “sensory anticipation” were primarily motivated by experiments that attempted to resolve fundamental questions about how the visual field manages to remain stable with the constant image-displacement introduced by physical activity, head and eye movements, and blinking (62, 63). At about the same time as these models of primary vision were conceived, species-generalizable PRCs-to-light had been developed across several experimental organisms occupying different ecological and temporal niches within the biosphere (45, 64, 65). Ironically, study of the non-image forming visual system had produced a wealth of empirical data (not to mention the resounding image of the PRC itself) attesting to the brain's prediction-making capabilities and its raison d'être in reducing the ongoing discrepancies occurring between expectation and actual experience. Yet, it was in the field of perceptual vision research that inference, prediction, and information-seeking became topics of intense scrutiny and now look to embody cutting-edge algorithms for machine vision and artificial intelligence [e.g., (66)].

Organisms were pressured to evolve a circadian timekeeping system that could make predictions about the environment because, ultimately, an inability to do so meant life or death vis-à-vis finding food, staying temperature-regulated, and avoiding predators. While direct responses to light independent of such a timekeeping mechanism (e.g., masking) would effectively restrict animals to a nocturnal or diurnal niche (67), they would not be sufficient for preparing and optimizing vast, interconnected areas of organismal physiology for times-of-day when—for example—food might be most readily available and digested or sleep might be most biologically restorative. Regarding entrainment, we have lost sight of these stakes and the inferences that came along with them—namely, that photodetection mechanisms in the service of the circadian pacemaker are likely to be highly flexible in the light information they use to localize sunset or sunrise. Evolutionary pressure not only coaxed the advent of an entrainable clock but also created a race to the bottom for sunlight detection. Organisms who won-out were able to use the least amount of light information in the service of entrainment and could interpret that information accurately whether it resulted from consistent or erratic contact with sunlight. Successful entrainment did not require a prolonged “sitting” audience with midday or twilight and, for some animals, could be achieved (well-enough) within their natural habitats by a few minutes' exposure once or twice a day (68, 69).

## Dynamic Light and the Circadian Pacemaker

Research has established the lower floors of circadian photoentrainment in laboratory models such as *Drosophila* and mice (70–72). Data suggest that most animals can synchronize and maintain a stable phase relationship to a 12-h light-dark schedule with an irradiance of <1 nW/cm^2^ or with skeleton photoperiods consisting of ~11-h intervals of darkness bookended by a pair of 30-min light pulses simulating dusk and dawn (70–75). Despite the appreciation that entrainment requires little in the way of photic energy, there is still a lingering assumption among chronobiologists that light-induced phase shifting demands a relatively large energy investment to trigger complete resetting of endogenous rhythmicity. This perspective is best couched by the reciprocity hypothesis, which asserts that the size of any phase-shift is directly proportional to the time-integrated illuminance the pacemaker registers from a light signal (76, 77). However, extant data suggest that light's association with circadian timekeeping is more complicated.

Emission technology in the 20th and early 21st centuries rarely offered control systems equipped to deliver light in a rapid, intermittent, and multidimensional fashion, where all physical exposure variables could be manipulated at once in quick successive steps. Within the technology milieu, however, were movie/photography studio devices that could produce microsecond and millisecond xenon flashes at fixed frequencies. These devices are still used today in order to illuminate and visualize color in dark scenes (e.g., Metz Mecablitz and DynaLite units). Over the past 60 years, several investigators have also used them to examine the phase-shifting effects of flashes in organisms as diverse as *Drosophila*, hamsters, rats, mice, and humans (78–86). The findings collated from these studies have established that the metazoan circadian pacemaker responds to intermittent millisecond flashes with phase shifts comparable to those that would have been generated with continuous, uninterrupted light administration, provided that the stimuli are delivered at regular intervals every few seconds or each minute. What's more, each circadian hour of the subjective night is equally amenable to flash stimulation; xenon-flash PRCs have been compiled for the eclosion rhythm of *Drosophila pseudoobscura* (79) and the flight activity of the Schneider's roundleaf bat (*Hipposideros speoris*) (80, 81), and this patterned stimulation has proven effective in both the delay and advance zones of C57BL/6 mice (82), the most common mouse strain bred in biomedical science.

Employing electrophysiology amplifiers and LED Ganzfeld lamps, researchers have summarized a few other observations germane to the pacemaker's reaction to (sub)millisecond light. First, the energy-efficiency with which flashes phase-shift the clock are maximized by shortening exposure, with optimization accruing all the way down to at least 10 μs (87–89). Ergo, it is likely that the circadian system responds to instantaneous light contact, habituates immediately thereafter, and then cycles through a rapid re-sensitization process. Second, flashes that reset the clock do so with a combinatorial logic that integrates the responses of these flashes with shorter and longer episodes of light (87). This means that flashes do not require delivery in some invariant or artificial sequence (e.g., with a fixed pulse duration, metered along a specific frequency) to impact the circadian system's timekeeping (90, 91). Third, the action spectra for flash resetting of circadian rhythms follows the action spectra that's been documented for visible light. Analogous to broad-spectrum xenon flashes, narrowband blue and green LED flashes can operate as stand-ins for continuous blue/green light exposure (92). Finally, the lower energy bounds for photic induction of circadian resetting reside within the micro-to-nanojoule range (92).

These observations lend support to a model where the pacemaker creates wholistic representations of twilight—thereby predicting the timing of the next day's dawn and dusk—by intermittently sampling bits of photic information that strike the retina as an organism navigates its environment. Presumably, sampling is done in rapid succession by capturing snapshots of incident light, integrating these snapshots across seconds/minutes, and favoring this integration process for the parts of the day when the sun's movement in the sky will invoke the greatest rates of change in ambient illumination intensity and spectral composition (i.e., the 30–60 min of twilight perceived when the sun is ascending or descending the horizon). Under this scenario, the pacemaker's intermittent reading frame is (1) optimized with photic information in its most dynamic state, and (2) withstands stochastic changes in light quantity and quality transiently introduced by clouds, wind and atmospheric turbidity (e.g., light scattering from wind-borne particles, haze), and by the behavior of the organism itself as it moves back-and-forth underground or underneath a discontinuous awning of trees and green vegetation (93).

It is worth noting that this model of circadian photoreception recapitulates an important gestalt principle of the image-forming visual system referred to as *closure*, which describes the brain's ability to perceive objects as a whole in their completeness even when the objects appear in the visual field lacking one or more constituent parts (94). The image-forming brain is not a stickler for the discrepancies that arise in detecting and identifying figures when they are obstructed, appear at an alternative angle, or when constituent parts may be physically absent. It compensates for the lack of information, interpolates what is missing, makes (mostly) correct deductions about the object or person in front of it, and actively “re-creates” the image of it. Compression algorithms are applied as soon as light contacts the retina, continue their processing as the signal traverses the thalamus and visual cortex, and culminate as the signal breaches the visual streams (95–97). Gestalt principles of visual perception detail how the image-forming system creates structure—and structure within space—by default. Analogous “gestalt” principles might be valuable toward explaining how the pacemaker creates automatic representations of time using twilight as a palette and compression algorithms requiring operation only within the circumscribed circuitry binding the retina and SCN.

## Flashes, Cones, and Circadian Predictions About Twilight

Throughout millennia, the pacemaker has been conditioned to track light transitions enveloping sunrise and sunset. The aggregated literature on flash-induction of circadian resetting suggests that this timekeeping mechanism occurs intermittently, using but a quantum of contact with light, to set in motion an integrative process that results in the synthetic construction of a ~60-min episode of twilight from just milliseconds of photic information; integration might occur throughout all stages of signal transduction across the retina and retinohypothalamic tract (RHT), SCN, and SCN outputs. With an engram of this temporarily stored in the SCN circuitry, the representation is then consulted to orient the synchronization of the SCN's output signal to the rest of the brain and periphery, thus phase-locking the next day's physiology and behavior. The literature that has burgeoned from Van den Pol and Heller's original observations (82)—that the circadian pacemaker orients to sequenced intermittent light exposures and not just individual flashes of oversaturating light (78, 80)—was facilitated by co-opting studio equipment but has since been enabled by the advent of semiconductor LEDs. These luminaires provide spectral/temporal photoemission control at the microsecond-level and offer the prospect of engineering dynamic patterns of flash exposure that quickly (and repeatedly) transition from one set of physical-exposure variables to another (98, 99). Despite this technology breakthrough, which is now ushering a fundamental shift in lighting practices around the world, many investigators still regard flash induction of circadian resetting as a lab curiosity—an example of a “circadian visual illusion” worth noting but not necessarily formalizing within studies of circadian photoreception (100). Perhaps what is needed is a better mechanistic characterization of the phenomenon and identification of the relative importance of each photoreceptor class to these types of physiological responses.

Prior to any experiment, one might hypothesize that classic photoreceptor cells would be important conduits for transducing flashes that will feed the pacemaker's twilight predictions. Both rods and cones contribute to RHT responses at light onset and provide short-latency inputs to ipRGCs. If the relative contribution of rods vs. cones were weighed, however, more data than not would suggest that cones are the more relevant photoreceptor class for flash conveyance. Cones with overlapping ranges of wavelength sensitivity: (1) Are the chief contributors to retinal responses driven by short-duration light, generating transient signals that account for most of the RHT activity evoked by a series of brief light pulses (8, 17–19, 33, 40–43); (2) Are disproportionately responsible for the upstream ability of the SCN to track one or more sudden fluctuations in light intensity and spectral contrast (e.g., akin to the salient changes in ambient illumination that characterize dawn and dusk) (33, 42, 44); (3) Differ from melanopsin or rods in that cones cannot drive sustained RHT activity during continuous, uninterrupted light exposure; accordingly, they are neither necessary nor sufficient for photoentrainment of behavioral activity rhythms to recurring solar or electric light-dark cycles (where light is presented in a relatively unwavering fashion for 10–12 straight hours) (27, 29, 33–37, 42); and (4) Are better than melanopsin or rods at adjusting their sensitivity to background illuminance (101–103), thus enabling them to operate as twilight detectors irrespective of how bright or long the photoperiod feeding into dusk. In short, cones are not “circadian-alignment” tools à la melanopsin or rods, which signal the enduring presence of light with fidelity thereby marking the day from the night and providing an estimation of daylength. Rather, they are critical sentinels for detecting the flickering kinds of light that signal the initiation of a sunset or sunrise has migrated to a time later or earlier than anticipated. In this role, they might or might not work together with rods, which already have established roles supporting photoentrainment and quantitative assessments of irradiance alongside melanopsin (10, 11, 27, 29, 42, 104).

**Figure 1:**
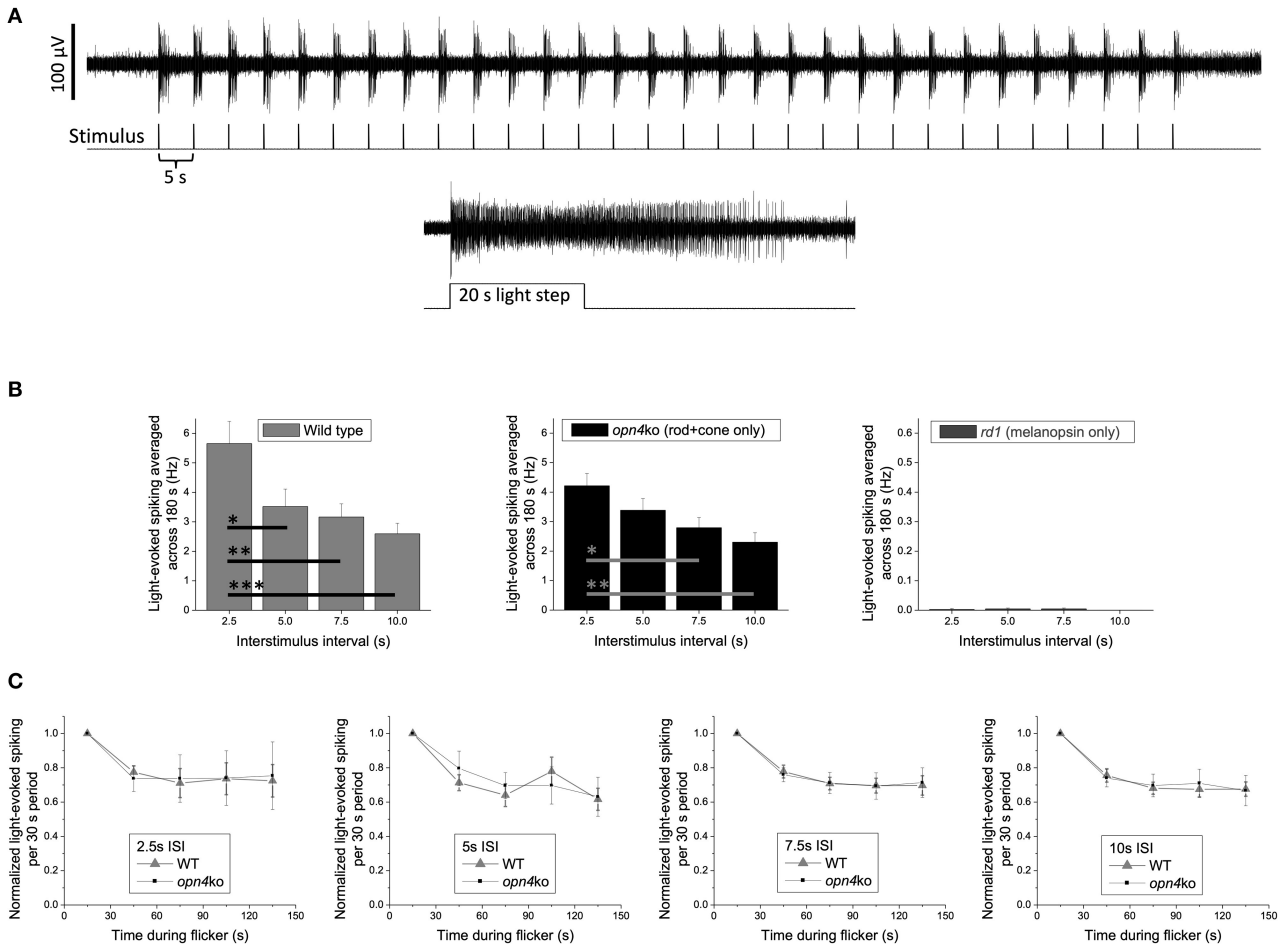
Spiking responses of mouse ipRGCs to a 150 s train of 2 ms flashes (2.5–10 s intervals) are mediated almost exclusively by rod/cone input. **(A)** Example response of a wild-type ipRGC to 30 flashes with a 5 s interstimulus interval (upper recording). This cell was identified as an ipRGC by its sustained response to a subsequent prolonged light step (lower recording), which evokes transient responses in all non-intrinsically photosensitive RGCs [Wong 2012; (35)]. Spike recording methods were identical to those described in the “Multielectrode-array recording” section in Wong 2012 except that all stimuli, including the 20 s light step, were 14.0 log photons cm^−2^ s^−1^ (~300 lux) full-field light produced by an LED with peak emission at 470 nm. **(B)** The light-induced elevation in spike rate averaged across the flicker plus 30 s of post-flicker darkness (to include responses outlasting the flicker) for C57BL/6 wild-type mice (*n* = 26 ipRGCs; left plot), *Opn4*^*Cre*/*Cre*^ melanopsin-knockout mice originally created by Ecker et al. (105) (*n* = 24 ipRGCs; middle plot), and *Pde6b*^*rd*1/*rd*1^ rod/cone-degenerate mice (*n* = 43 ipRGCs; right plot); note the expanded y-axis in the right plot. All mice in each group were about 7 months old and included both sexes. Though flashes delivered with a 2.5 s interstimulus interval appear to cause a greater spike rate increase in wild-type vs. melanopsin-knockout mice, this difference is not statistically significant (*p* = 0.156). The wild-type vs. melanopsin-knockout differences for the other three intervals are also statistically insignificant (*p*-values between 0.689 and 0.818), suggesting that melanopsin does not enhance the flicker responses. Asterisks represent *p*-values calculated using one-way ANOVA with *post-hoc* Tukey test: *, *p* < 0.05; **, *p* < 0.01; ***, *p* < 0.001. **(C)** For all four interstimulus intervals, the gradual decay in light-evoked spike rate during the 150 s flicker is comparable between wild-type and melanopsin-knockout mice, suggesting that melanopsin does not contribute to any temporal summation of successive flash responses. Error bars are S.E.M.

Retinally degenerate and knockout mice offer powerful platforms for gauging the relative contribution of each photoreceptor class to a clock light-response of interest. As a proof-of-concept test of our suggestion that classic photoreceptors mediate the circadian effects of millisecond light flashes, one of the authors (KYW) recorded *ex vivo* ipRGC responses from *Opn4*^*Cre*/*Cre*^ melanopsin-knockout (105) vs. *Pde6b*^*rd*1/*rd*1^rod/cone-degenerate mice. The stimuli were 150-s trains of 2-ms flashes (full-field; 470 nm, 14.0 log photons cm^−2^ s^−1^ or roughly 300 lux) with various interstimulus intervals (ISI, 2.5–10 s; Figure 1). These experiments were motivated by previous observations suggesting that: (1) Melanopsin responds better to 20 1-s flashes with certain ISIs than to a continuous 20-s light step, indicating temporal summation (106); and (2) Flashes 50-ms in duration could, occasionally, evoke a melanopsin response in ipRGCs (34), indicating that melanopsin might respond to short as well as prolonged illumination. Given these properties, and the lack of any preexisting data on temporal summation of shorter flashes (i.e., <1 s) by melanopsin, it was not immediately obvious that there would be a clear dichotomy between an ipRGC's outer retinal photoreceptor-driven and melanopsin-mediated responses to millisecond flash stimulation. Such a dichotomy did emerge, however. Upon flash exposure, wildtype mouse ipRGCs with fully-intact rod/cone input showed increases in ipRGC spiking that scaled inversely with ISI (Figure 1B, left panel). Melanopsin-deficient ipRGCs (*Opn4*^*Cre*/*Cre*^) exhibited similar ISI-dependent patterns of flash response, but ipRGCs in retinas largely devoid of rods and cones (*Pde6b*^*rd*1/*rd*1^) mounted virtually no response (Figure 1B, middle and right panels, respectively). Subsequent head-to-head analysis of wildtype and *Opn4*^*Cre*/*Cre*^ ipRGC light-evoked spiking indicated that the decay in the spike rate over the stimulation window was not statistically different for any of the ISI conditions, suggesting that melanopsin did not contribute to any temporal summation of the 2-ms flashes (Figure 1C).

Like all data collected from retinal-degenerate mice, these results need to be interpreted with caution. These models do not allow us to visualize what would occur within an intact system where all photoreceptor classes influence the activity of one another, nor control for the possibility of compensatory reorganization of the circuit loops in which these photoreceptors operate [e.g., (107)]. There is also the caveat that melanopsin might have responded to flashes had they been delivered with higher-intensity stimulation protocols [moderate regimens were tested here, instead, because they are more physiologically relevant for nocturnal rodents and better conform to the flash intensities that have been studied in humans; (91)]. All that said, the clarity of the data and the conclusion they offer remain striking: rods and/or cones enable ipRGC detection and integration of light flashes independent of melanopsin, whose contribution—if any—is predicated on initial processing by the outer retinal photoreceptors. The results are consistent with the suppositions made in the current review and with the idea that cones (with or without the input of rods) will prove to be key regulators of flash-induced resetting of pacemaker rhythms. Future experiments using mice with intact retinal circuitry (e.g., *Opn1mw*^*R*^) or with selective loss in rod vs. cone photosensitivity [e.g., *Gnat1*^−/−^ and *Gnat2*^*cpfl*3^ mice (108–110)] will be necessary to isolate the relative contributions of cones to this phenomenon and to determine whether the retina processes staccatos of narrowband light with any circadian phase-dependence. Using principles of silent substitution, flash stimuli differing in the amount of cone-and-rod excitation might also be probed for their circadian effects in retinally-intact humans compared to humans with congenital achromatopsia (i.e., people without a functional cone system; prevalence one in 30,000–50,000) (111).

## Conclusion

The way we illuminate our world is changing, moving away from unidimensional forms of illumination provided by gas-discharge fluorescent lamps to highly customizable solid-state lighting with semiconductor LEDs. Data suggest that this watershed is material to understanding the circadian pacemaker's phase-responses to electric light exposure, which are likely rooted within a natural process where the pacemaker “flash-samples” the photic information available at twilight to create predictions about the timing of the next day's dusk and dawn. While the mechanisms subserving flash photoreception and the SCN's twilight predictions require further study, it is becoming clear that classical photoreceptors, including cones, operate as sensors in this process. The “Cone Sentinel Model” we articulate here [and hinted at by Zeitzer; (91)] raises many considerations for how different combinations of narrowband LED stimulation can be strung together in phototherapy protocols to improve mental and physical health (112). The gestalt inferences made in this model will be challenging to demonstrate experimentally. However, prudent first steps might include the design of studies examining the differential phase-shifting effects of flashes patterned after twilight progressions—vs. more randomly generated sequences—in parallel to those examining the neurophysiological responses arising from flash regimens highly-optimized (or ill-suited) for driving cone input to ipRGCs.

## Data Availability Statement

The original contributions generated for this study are included in the article/supplementary material, further inquiries can be directed to Kwoon Y. Wong, kwoon@umich.edu.

## Ethics Statement

The animal study was reviewed and approved by the University of Michigan Institutional Animal Care and Use Committee (IACUC).

## Author Contributions

F-XF conceptualized the Cone Sentinel model, which was further refined by KYW. KYW developed and carried out the retinal electrophysiology experiments. All authors contributed to writing the manuscript.

## Conflict of Interest

The authors declare that the research was conducted in the absence of any commercial or financial relationships that could be construed as a potential conflict of interest.
